# Structure and Specificity of the Bacterial Cysteine Methyltransferase Effector NleE Suggests a Novel Substrate in Human DNA Repair Pathway

**DOI:** 10.1371/journal.ppat.1004522

**Published:** 2014-11-20

**Authors:** Qing Yao, Li Zhang, Xiaobo Wan, Jing Chen, Liyan Hu, Xiaojun Ding, Lin Li, Jayashree Karar, Hongzhuang Peng, She Chen, Niu Huang, Frank J. Rauscher, Feng Shao

**Affiliations:** 1 National Institute of Biological Sciences, Beijing, China; 2 The Wistar Institute, Philadelphia, Pennsylvania, United States of America; 3 National Laboratory of Biomacromolecules, Institute of Biophysics, Chinese Academy of Sciences, Beijing, China; 4 National Institute of Biological Sciences, Beijing, Collaborative Innovation Center for Cancer Medicine, Beijing, China; Collège de France, France

## Abstract

Enteropathogenic *E. coli* (EPEC) and related enterobacteria rely on a type III secretion system (T3SS) effector NleE to block host NF-κB signaling. NleE is a first in class, novel *S*-adenosyl-L-methionine (SAM)-dependent methyltransferase that methylates a zinc-coordinating cysteine in the Npl4-like Zinc Finger (NZF) domains in TAB2/3 adaptors in the NF-κB pathway, but its mechanism of action and other human substrates are unknown. Here we solve crystal structure of NleE-SAM complex, which reveals a methyltransferase fold different from those of known ones. The SAM, cradled snugly at the bottom of a deep and narrow cavity, adopts a unique conformation ready for nucleophilic attack by the methyl acceptor. The substrate NZF domain can be well docked into the cavity, and molecular dynamic simulation indicates that Cys673 in TAB2-NZF is spatially and energetically favorable for attacking the SAM. We further identify a new NleE substrate, ZRANB3, that functions in PCNA binding and remodeling of stalled replication forks at the DNA damage sites. Specific inactivation of the NZF domain in ZRANB3 by NleE offers a unique opportunity to suggest that ZRANB3-NZF domain functions in DNA repair processes other than ZRANB3 recruitment to DNA damage sites. Our analyses suggest a novel and unexpected link between EPEC infection, virulence proteins and genome integrity.

## Author Summary

Pathogens often manipulate host functions by posttranslational modifications such as ubiquitination and methylation. The NF-κB pathway is most critical for immune defense against infection, thereby frequently targeted by bacterial virulence factors. NleE, a virulence effector from EPEC, is a SAM-dependent methyltransferase that modifies a zinc-finger cysteine in TAB2/3 in the NF-κB pathway. NleE is not homologous to any known methyltransferases. We present the crystal structure of SAM-bound NleE that shows a novel methyltransferase fold with a unique SAM-binding mode. Computational docking and molecular dynamics simulation illustrate a structural and chemical mechanism underlying NleE recognition of the NZF and catalyzing site-specific cysteine methylation. Subsequent substrate specificity analyses identify an N-terminal region in TAB3 required for efficient NleE recognition as well as another NZF protein ZRANB3 being a new substrate of NleE. NleE-catalyzed cysteine methylation also disrupts the ubiquitin chain-binding of ZRANB3-NZF domain, providing new insights into ZRANB3-NZF functioning in DNA damage repair. These results reinforce the idea of harnessing bacterial effectors as a tool for dissecting eukaryotic functions.

## Introduction

NF-κB signaling plays a central role in defending against bacterial infection [1], [2]. The NF-κB signaling initiates innate immune responses and inflammation via a myriad of pathogen-recognition or cytokine receptors. These receptors generate ubiquitin-chain signals that are directly recognized by the TAB2/3 adaptors, thereby activating the TAK1 and IKK kinase cascade, leading to transcription of genes involved in immune defense. EPEC and the related enterohaemorrhagic *E. coli* (EHEC) block NF-κB signaling using virulence effector proteins injected into host cells by the type III secretion system (T3SS). The NleE effector, conserved in *Shigella* and *Salmonella*, plays a major role in EPEC suppression of the NF-κB signaling in cell culture infection [3], [4], [5]. We recently discovered that NleE is a SAM-dependent methyltransferase that modifies a cysteine in the NZF domains of TAB2/3, thereby disrupting ubiquitin-chain sensing of TAB2/3 and abolishing NF-κB-mediated proinflammatory responses [6].

Protein methylation is of great importance in a plethora of cellular processes including biosynthesis, signal transduction, protein repair, chromatin regulation and gene silencing [7]. SAM-dependent methyltransferases are diverse in their primary sequence, three dimensional structure and SAM-binding mode, and have been classified into five different families (Class I-V) [8]. The five families of methyltransferases generally catalyze lysine or arginine methylation. NleE-catalyzed cysteine methylation of TAB2/3 is the first example of enzyme-catalyzed protein cysteine methylation, representing a novel mechanism in regulating signal transduction in eukaryotes. NleE harbors no sequence homology to known methyltransferases. The structural basis for NleE methyltransferase activity and substrate specificity are unknown.

Here we determine the crystal structure NleE-SAM complex, which reveals a novel methyltransferase fold and a unique mode of SAM binding. Molecular dynamic simulation of the docked NleE-SAM-NZF complex indicates that Cys673 in TAB2-NZF is structurally and energetically favorable for attacking the SAM. Profiling of a large number of zinc fingers identifies ZRANB3 as a new NleE substrate. ZRANB3 is recruited to damaged DNA replication forks and functions in maintaining genome integrity [9], [10], [11]. NleE-methylated ZRANB3-NZF domain lost the ubiquitin chain-binding activity, suggesting an unexpected link between EPEC infection, virulence proteins and genome integrity. These structural and functional analyses suggest that NleE may target ZRANB3 or other zinc-finger proteins for cysteine methylation in promoting bacterial virulence.

## Results

### NleE targets TAB2/3-NZFs for cysteine methylation *in vivo*

**Figure 1:**
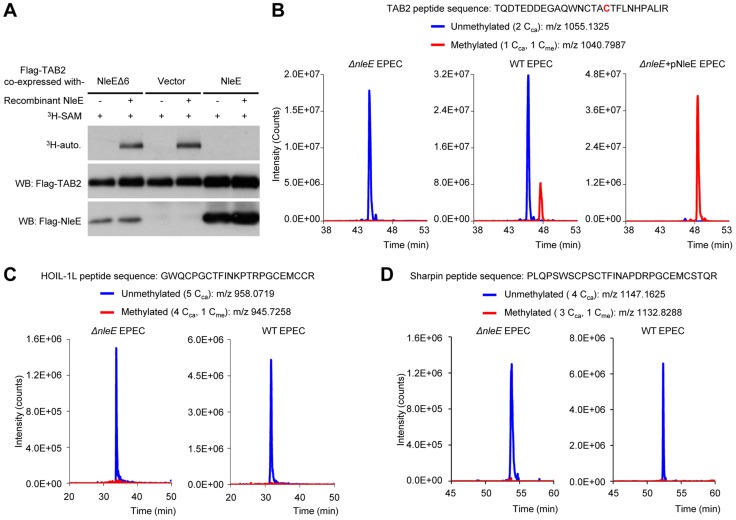
NleE specifically targets TAB2/3 in the NF-κB pathway. (A) Back-methylation analysis of NleE modification of cellular TAB2. Flag-TAB2 co-expressed with a vector or NleE in 293T cells was immunopurified and incubated with recombinant NleE in the presence of ^3^H-SAM. NleEΔ6, NleE mutant with deletion of _209_IDSYMK_214_. (B–D) Mass spectrometry analysis of NleE modification of TAB2 (B), HOIL-1L (C) and Sharpin (D) during EPEC infection. Shown are the extracted ion chromatograms of triply charged NZF-domain peptides from TAB2, HOIL-1L-V195R or Sharpin-S346R purified from infected 293T cells. The unmethylated peptides are shown in blue trace and the methylated ones are in red with the methylated cysteine residue in red. The V195R and S346R mutations were introduced to facilitate mass spectrometry identification of the tryptic peptides. C_ca_, carbamidomethylated cysteine generated from iodoacetamide treatment during sample preparation; C_me_, NleE-methylated cysteine.

Due to the lack of an antibody capable of recognizing methylated cysteine, we developed a back-methylation assay by examining the sensitivity of TAB2 purified from NleE-transfected mammalian cells to *in vitro* re-methylation by NleE (Figure 1A). Flag-TAB2 from 293T cells was efficiently re-methylated, whereas that from cells co-transfected with wild-type NleE resisted further *in vitro* methylation by NleE, suggesting full methylation of cellular TAB2 by transfected NleE. Furthermore, tandem mass spectrometric analysis of TAB2/3 from infected 293T cells confirmed the methylation modification as a Cys673-methylated peptide from Flag-TAB2 was detected upon infection with wild-type EPEC but not the Δ*nleE* strain (Figure 1B). Complementation of Δ*nleE* strain with NleE expressed from a high-copy plasmid resulted in complete methylation of Cys673 (Figure 1B). This provides direct evidences that NleE carries out cysteine methylation of TAB2/3 during EPEC infection. In addition to TAB2/3, components of the linear ubiquitin chain assembly complex (LUBAC), HOIP, HOIL-1L and Sharpin, also contain NZF domains and play important roles in NF-κB signaling [12], [13], [14]. Consistent with our previous *in vitro* data, the HOIL-1L and Sharpin-derived tryptic peptides bearing the cysteine corresponding to Cys673 in TAB2 were not methylated even when the infection was performed with the NleE-proficient EPEC strain (Figure 1C and 1D). These data support that NleE inhibition of NF-κB signaling results from its specific targeting TAB2/3-NZF domains for cysteine methylation.

### Overall structure of NleE-SAM complex

To understand the mechanism of NleE function, we attempted to determine its crystal structure. Wild-type NleE yielded poor crystals, but NleE K181A protein, out of 15 Lys-to-Ala mutants designed to improve the crystallization [15], produced sufficient-quality crystals in the C2 space group. A model obtained from 2.6-Å diffraction data collected on the selenomethionine (Se-Met) protein was further refined and a final resolution of 2.3 Å was achieved (Table S1). The structure shows that the mutated K181A is exposed and located at the interface of crystal contacts (Figure S1). Despite the presence of four NleE in an asymmetric unit (chain A–D) (Figure S2A), NleE was exclusively a monomer in solution as judged by gel filtration chromatography analysis. The size of the buried surface area formed between different chains ranged from 590 Å^2^ to 1722 Å^2^ (Figure S2B). PISA (http://www.ebi.ac.uk/pdbe/pisa/) analysis of protein interface present in the crystal also suggested that the NleE tetramer is unlikely to be stable in solution (Figure S2C), indicating that the tetrameric assembly of NleE in the asymmetric unit results from crystal packing likely with no physiological relevance. No meaningful structural difference was found among the four molecules and therefore only chain A was analyzed hereinafter.

**Figure 2:**
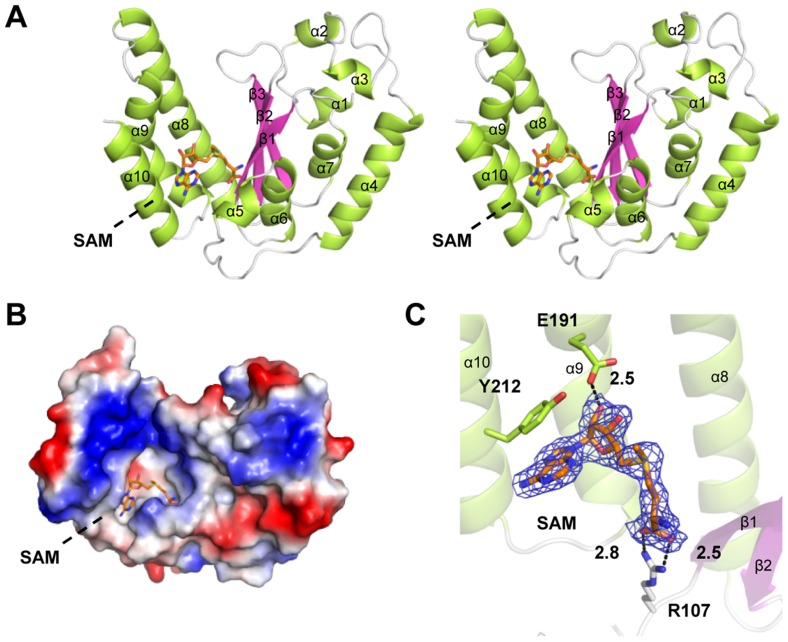
Crystal structure of NleE in complex with SAM. (A) Stereoview of NleE structure in ribbon diagram with green α-helices, magenta β-strands and grey loops. SAM is in orange sticks. Secondary structure elements are labeled in sequential numbers (α1–10, β1–3). (B) Electrostatic surface potential of NleE structure. The color scale from red (−5 kT/e) to blue (5 kT/e) was calculated in PyMol. (C) A close view of the SAM binding core. SAM is meshed with σ-A weighted Fo-Fc simulated annealing omit map contoured at 3.0σ. SAM interaction residues (Arg107, Glu191 and Tyr212) are also in sticks. Polar interactions are represented by black dashed lines with a number denoting the distance in angstrom. The color scheme follows that in (A).

The structure of NleE (residues 21–220: residues 1–20 and 220–224 lacked electron density) adopts α/β doubly wound topology with a central three-stranded anti-paralleled *β*-sheet (β1–β3) sandwiched by α-helices (*α*1–α10) (Figure 2A). β1–β3 are arranged in the left-right-middle order, which, together with the flanking α-helices (α8–α10), generates a deep and narrow cavity on the left side of NleE. The SAM molecule fits snugly into the cavity (Figure 2B), which buries 2374 Å^2^ solvent accessible surface area, corresponding to 76% of the total surface area of SAM and leaving the rest of 24% exposing to the solvent.

### Arg107, Glu191 and Tyr212 bind to the SAM and are important for NleE function

We first analyzed the structural details of SAM binding in NleE. The interior of the SAM-binding cavity is filled with hydrophobic side chains, but polar interactions appear to play a key role in riveting the SAM into the cavity (Figure 2C). Specifically, the α-carboxylic group of the amino acid moiety of the SAM is coordinated by the side chain of Arg107 situated on a loop connecting β1 and α6. The hydroxyl group of the ribose of the SAM is hydrogen-bonded with the side-chain carboxylic group of Glu191. The adenine ring of SAM is oriented by the aromatic ring of Tyr212 residing at α10 (residues 206–219) through a π-π stacking interaction. This explains the complete functional loss of the NleEΔ6 mutant [4], [6] as deletion of _209_IDSYMK_214_ is expected to disrupt the α10. The interactions embed the SAM at the bottom of the cavity with a narrow opening slit, through which the buried ligand presents its methylthio in a direction favorable for the S_N_2 methyltransfer.

**Figure 3:**
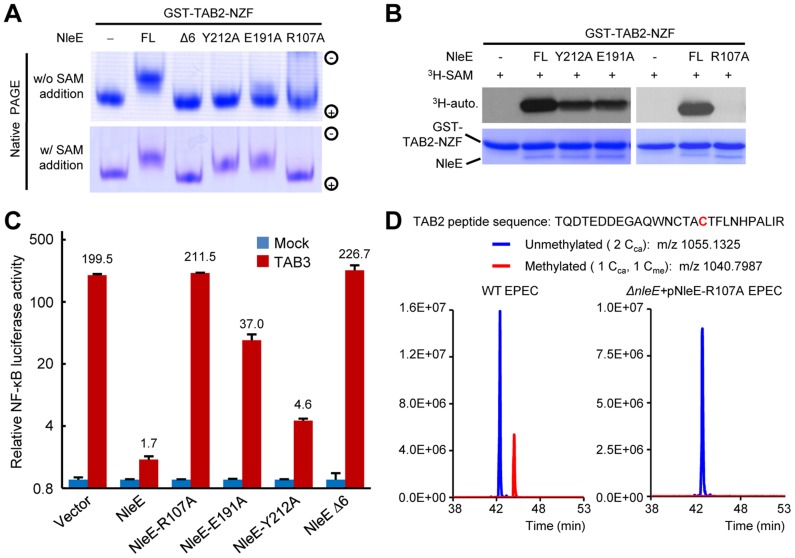
Functional analyses of NleE SAM-binding mutants. (A, B) Effects of SAM-binding site mutations on methylation of TAB2-NZF. GST-TAB2-NZF was incubated with NleE or indicated mutants in the absence (upper) or presence (lower) of exogenous SAM, followed by native-PAGE analysis (A). ^3^H-SAM was added into the reaction and the reaction was analyzed by SDS-PAGE electrophoresis in (B). FL indicates the full-length NleE. (C) Luciferase assays of the activity of NleE mutants in blocking the NF-κB signaling. 293T cells were transfected with TAB3 together with an indicated NleE variant. The Y axis is on the logarithmic scale. Error bar, standard deviation. (D) Mass spectrometry analysis of NleE-R107A mutant modification of TAB2 during EPEC infection. Shown are the extracted ion chromatograms of triply charged NZF-domain peptides from TAB2 purified from infected 293T cells. The unmethylated peptides are shown in blue trace and the methylated ones are in red with the methylated cysteine residue in red. C_ca_, carbamidomethylated cysteine generated from iodoacetamide treatment during sample preparation; C_me_, NleE-methylated cysteine.

Mutation of Arg107, Glu191 or Tyr212 in NleE all abolished *in vitro* methylation of GST-TAB2-NZF, revealed by native gel mobility analysis of the NZF domain (Figure 3A). When exogenous SAM was added, NleE-E191A and Y212A showed activity equal to wild-type NleE, whereas NleE-R107A and NleEΔ6 remained completely inactive (Figure 3A). Consistently, NleE-Y212A and NleE-E191A were partially impaired in transferring ^3^H-methyl from radiolabeled SAM onto TAB2-NZF; NleE-R107A showed absolutely no activity in this assay (Figure 3B). Thus, Arg107 is most critical for SAM binding while NleE-Y212A and NleE-E191A are severely impaired. The *in vivo* activity of NleE-R107A, E191A or Y212A mutants in inhibiting NF-κB in transfected 293T cells was concordant with their *in vitro* methylation activity (Figure 3C). Further supporting the structural analysis, the NleE-R107A mutant, when complemented into EPEC Δ*nleE* strain, failed to restore methylation of TAB2 in bacteria infected cells (Figure 3D).

### Structural comparison of NleE and its SAM-binding mode with other methyltransferases

**Figure 4:**
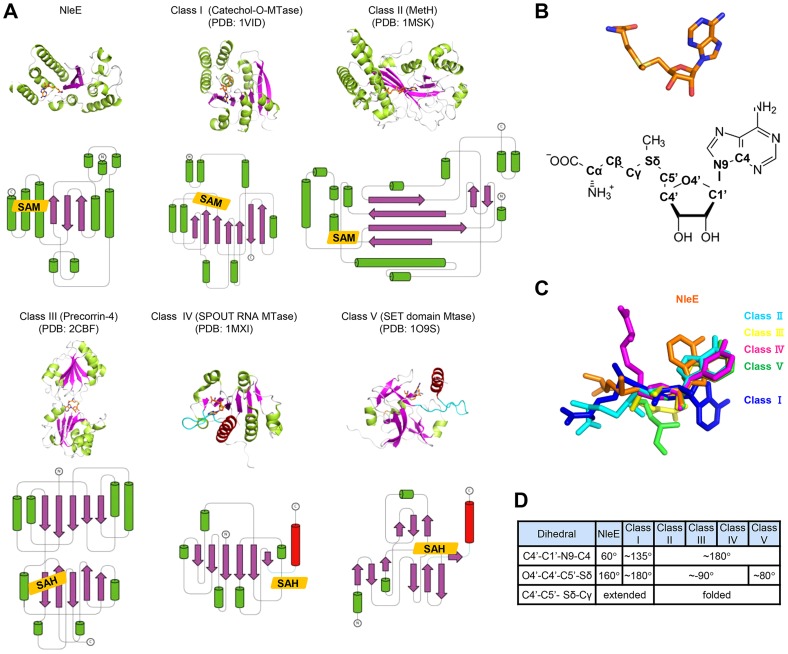
Structural comparison of NleE-SAM complex with other SAM-dependent methyltransferases. (A) Representative methyltransferase structures. The upper and lower panels show the ribbon diagram and the corresponding topology, respectively. α-helices, β-sheets and loops are in green, magenta and grey, respectively. SAM/SAH is shown as orange sticks in the ribbon diagram and orange rhombus in the topology diagram. The knot α-helixes in Class IV and V methyltransferases are in red and the linking loops are drawn as cyan lines. (B) The conformation of SAM in NleE (upper) and its atomic nomenclature (lower). (C) Structural alignment of SAM in NleE with that in other methyltransferases using the ribose ring as the reference. (D) The conformational difference between NleE-bound SAM and SAMs in other five classes of methyltransferases. SAM conformation is denoted by three dihedral angles indicated in the table. The atomic nomenclature of SAM follows that in (B).

Among the five families of SAM-dependent methyltransferases [8], the most abundant Class I has a central seven-stranded β-sheet and a GxGxG SAM-binding motif; Class II has long β-strands and a shallow groove with a RxxxGY SAM-binding motif; Class III is a homodimer with each monomer adopting an αβα structure and the SAM moiety bound between the two monomers; Class IV (the SPOUT family of RNA methyltransferases) bears a C-terminal SAM-binding knot structure; Class V contains a SET-domain SAM binding motif composed of three small β-sheets. Remarkably, the overall architecture of NleE does not resemble any of the five families (Figure 4A), thus representing a completely novel class of methyltransferases. Moreover, the conformation of the SAM in NleE is much different from other methyltransferases as reflected in the adenosine and methionine conformations (Figure 4B and 4C).The adenine base in NleE-bound SAM is characterized by a C4′-C1′-N9-C4 dihedral angle of 60°, significantly smaller than that in other methyltransferase-bound SAM or *S*-adenosylhomocysteine (SAH) (Figure 4D). The O4′-C4′-C5′-Sδ dihedral angle in NleE-bound SAM is 160°, comparable to the ∼180° in that in Class I methyltransferases, whereas this dihedral angle is approximately −90° in Class II-IV and 80° in Class V methyltransferases (Figure 4D). According to the C4′-C5′-Sδ-Cγ dihedral angle, the SAM/SAH molecules in NleE, Class I and II methyltransferases adopt a relatively extended conformation while those in other three classes adopt a more compact structure (Figure 4C).

### Cys673 in TAB2-NZF is structurally favorable for methyl transfer from NleE

**Figure 5:**
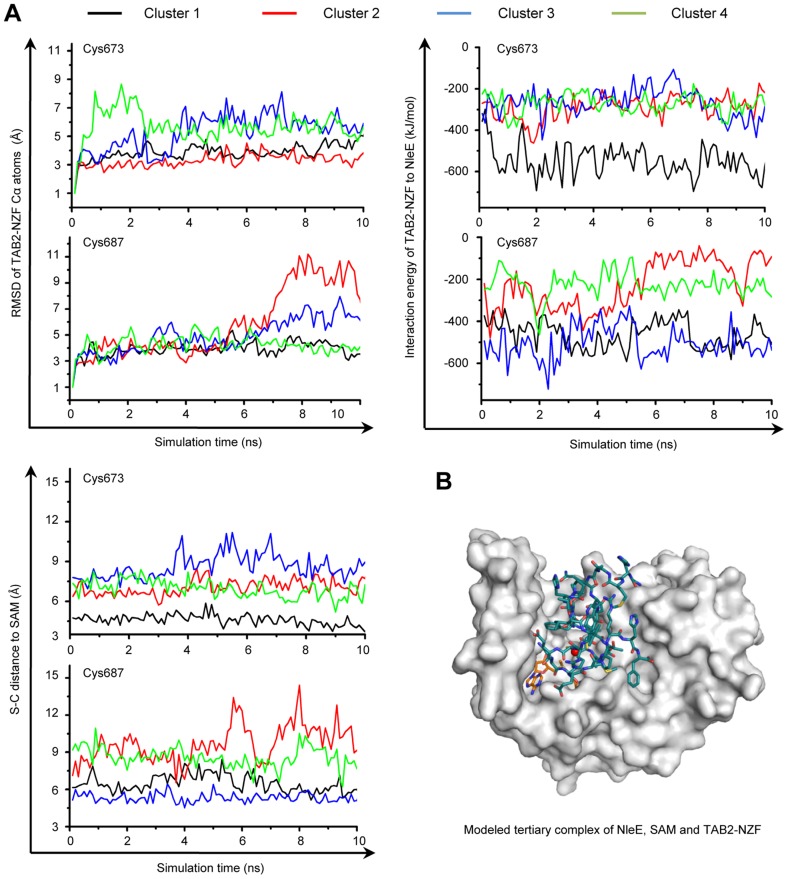
MD simulation shows Cys673 in TAB2-NZF being structurally favorable for methyltransfer from NleE. (A) The dynamic motion (indicated by the RMSD values) (upper left), the change of interaction energy (upper right) and the S-C distance (lower left) of the largest four clusters in Cys673 or Cys687-restrainted 15-ns MD simulation (shown are snap shots of the last 10-ns simulation). Representative poses of the four clusters (black, red, blue and green curve) obtained from protein docking performed with restrained distance from Cys673/Cys687 to the methyl carbon in SAM (S-C distance) were used as starting templates for the 15-ns MD simulation shown. (B) Overall view of the docked NleE-SAM-NZF complex. NleE is shown as white surface model; the SAM is in orange sticks; TAB2-NZF is in blue sticks with the Zn represented by a red dot.

NleE specifically methylates Cys673 in TAB2 (Cys692 in TAB3) among the four Zn-coordinating cysteines in TAB2/3-NZF domains despite that they are predicted to be chemically inert due to protection by hydrogen bonds [16] (Figure S3A). In the TAB2-NZF structure (PDB ID code: 3A9J), Cys673 and Cys687 are largely exposed, whereas Cys670 and Cys684 are completely buried (Figure S3B). To understand the mechanism of site-specific methylation by NleE, a hierarchical protein-protein docking approach with enforced distance restraints between the methyl group of SAM and the sulfur of Cys673/Cys687 was employed and molecular dynamics (MD) simulation was performed. The Cys687-restricted simulation showed a dramatic motion with pronounced root-mean-square deviation (RMSD) values, high interaction energy and a large distance from Cys687 to the methyl donor (Figure 5A and Figure S3C). In contrast, the Cys673-restricted simulation showed a relatively limited motion and lower energy with a close distance from Cys673 to the methyl donor. Thus, a most energetically favorable and structurally stable NleE-SAM-NZF complex was *in silico* modeled (Figure 5B), which clearly showed that Cys673 is the most favorable substrate residue.

### Efficient substrate recognition requires an N-terminal region in TAB2/3

**Figure 6:**
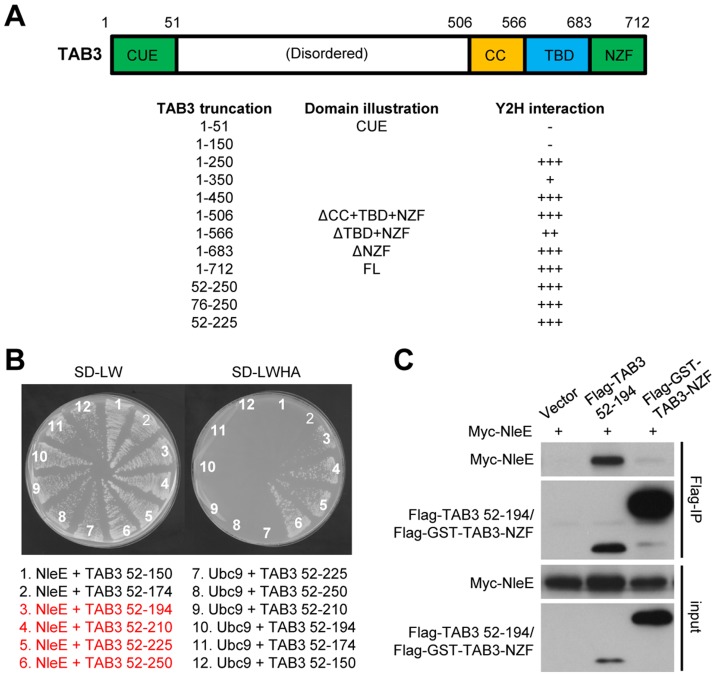
NleE binds to an N-terminal region in TAB2/3 for efficient recognition. (A) Domain illustration of TAB3 and summary of NleE interaction with TAB3 truncations. Interaction strength is scored by the number of + (also see the related data in Figure S4). CUE, CUE ubiquitin binding domain; TBD, TAK1-binding domain; CC, coiled-coil domain; NZF, Nlp4-like zinc finger domain. (B) Yeast two-hybrid assay of NleE interaction with TAB3 truncations. Yeast strain AH109 was transformed with plasmid combinations as illustrated (bait+prey). Plasmid combinations resulting in a positive interaction are highlighted in red. (C) Co-immunoprecipitation interaction assay of NleE and TAB3 truncations in transfected 293T cells. Shown are immunoblots of anti-Flag immunoprecipitates (Flag IP) and total cell lysates (Input).

We previously observed that deletion of the NZF from TAB3 (TAB3ΔNZF) does not affect its binding to NleE [6] (Figure 6A, and Figure S4A and S4B). This suggested that specific recognition by NleE requires another region in TAB3. Progressive truncations from both the C and N termini of TAB3ΔNZF identified residues 52–194 as the minimal fragment sufficient for binding to NleE in the yeast two-hybrid interaction assay (Figure 6A and 6B, and Figure S4). Co-immunoprecipitation assay in transfected 293T confirmed that residues 52–194 of TAB3, in contrast to the NZF alone, were competent in efficient binding to NleE (Figure 6C). Thus, binding of the N-terminal region in TAB3 (possibly also TAB2) may serve as a docking mechanism for recognition and methylation of TAB2/3-NZF by NleE. However, it is worth noting here that this region, involved in docking TAB3 onto NleE, does not appear to be sufficient for NleE methylating of other NZF domain as an NleE-resistant ZRANB2-NZF (see below) remained unmodified by NleE even when positioned in place of TAB3-NZF in the TAB3 ΔNZF construct (Figure S5).

### NleE methylates the NZF domain of ZRANB3 and disrupts its ubiquitin-chain binding

**Figure 7:**
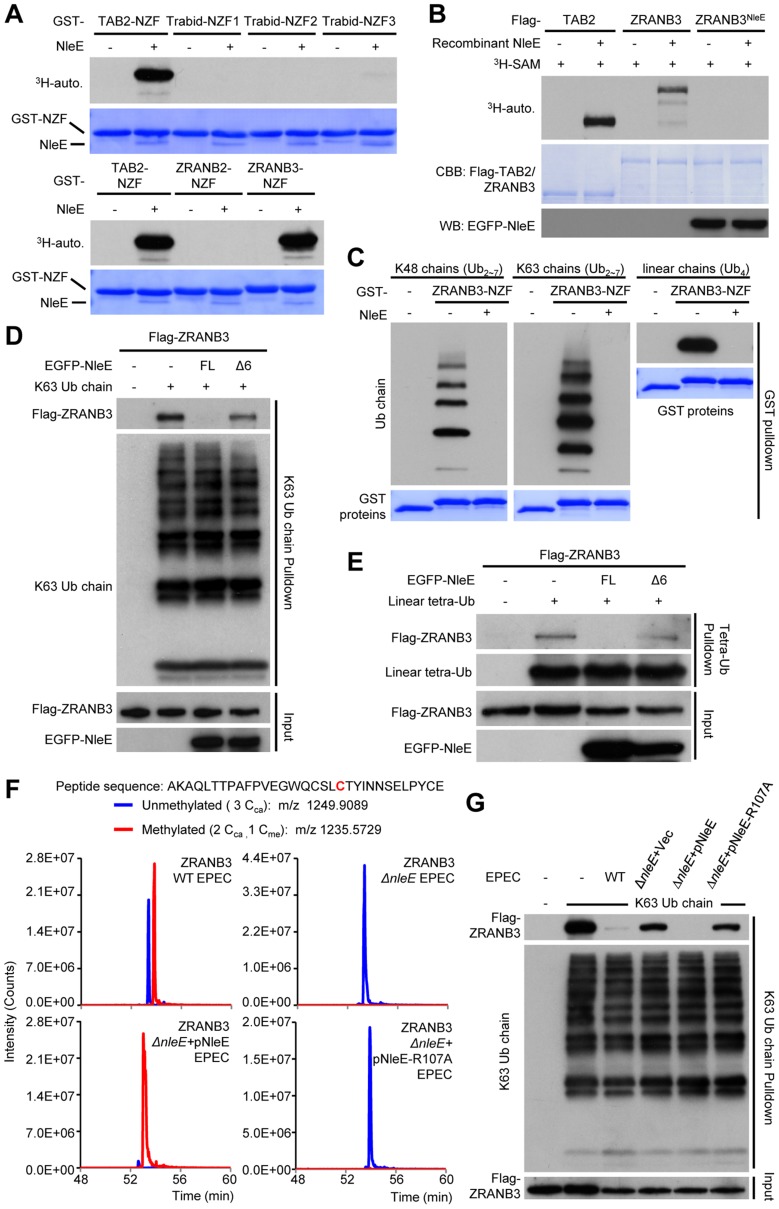
NleE targets ZRANB3-NZF domain for cysteine methylation and disrupts its ubiquitin-chain binding. (A) ^3^H-SAM labeling assay of NleE methylation of different GST-NZFs. (B) ^3^H-SAM labeling assay of NleE methylation of full-length Flag-TAB2/ZRANB3 purified from control or NleE-expressing 293T cells (Flag-ZRANB3^NleE^). (C–E) Assays of ubiquitin-chain binding of NleE-modified ZRANB3-NZF or full-length ZRANB3. Indicated ubiquitin chains were subjected to pulldown by purified GST-ZRANB3-NZF that was left untreated or had been incubated with recombinant NleE (C). Lysates of 293T cells transfected with Flag-ZRANB3 and NleE were subjected to pulldown by indicated ubiquitin chains (D and E). Shown are immunoblots of the pulldown (Ub chain pulldown) and total cell lysates (Input). FL indicates the full-length NleE. (F) Mass spectrometry analysis of NleE modification of ZRANB3 during EPEC infection. Shown are the extracted ion chromatograms of doubly charged NZF-domain peptides from ZRANB3 purified from infected 293T cells and digested by Glu-C. The unmethylated peptides are shown in blue trace and the methylated ones are in red with the methylated cysteine residue in red. C_ca_, carbamidomethylated cysteine generated from iodoacetamide treatment during sample preparation; C_me_, NleE-methylated cysteine. (G) Assays of ubiquitin-chain binding of NleE-modified Flag-ZRANB3 during EPEC infection. Lysates of 293T cells transfected with Flag-ZRANB3 and infected with indicated EPEC strain were subjected to Lys63-linked ubiquitin-chain pulldown. Shown are immunoblots of the pulldown (K63 Ub chain pulldown) and total cell lysates (Input).

Given that Zn coordination is required for cysteine methylation by NleE, we investigated whether other Zn fingers could also be a substrate of NleE. Among a total of more than 50 Zn fingers including C2H2, RING, RBCC/TRIM, FOG, PHD, as well as all the 13 NZF C4 fingers (Table S2 and Figure S6), NleE efficiently methylated the NZF domain of ZRANB3 with similar efficiency to that of the NZF domains of TAB2/3 and yeast Vps36 (Figure 7A). NleE did not modify Npl4, Sharpin, HOIP, HOIL-1L, Trabid-NZF1/2/3 and ZRANB2-NZF among the NZF subfamily [6] (Figure 7A and Table S2). Tandem mass spectrometry analysis identified the second cysteine in ZRANB3 (Cys630) being the methylation site, which echoes the situation with TAB2/3-NZF domains (Figure S7). Full-length ZRANB3 purified from 293T cells was also a robust substrate in the in vitro methylation assay (Figure 7B). In the back-methylation assay, recombinant NleE failed to methylate ZRANB3 purified from NleE-expression 293T cells (Figure 7B), suggesting a full methylation of ZRANB3 in transfected mammalian cells. Agreeing with that reported in previous studies [9], [11], GST-ZRANB3-NZF could bind to polyubiquitin chains of Lys63, Lys48, as well as tetra ubiquitin with linear linkage (Figure 7C). However, methylation by NleE was found to abolish the binding of ZRANB3-NZF to all ubiquitin chains. NleE could also abolish the ubiquitin chain binding of full-length ZRANB3 in transfected 293T cells, whereas the methyltransferase-deficient NleEΔ6 mutant failed to do so (Figure 7D and 7E). In EPEC-infected cells, the majority of Flag-ZRANB3 appeared to be methylated in an NleE-dependent manner (Figure 7F). The diminished ZRANB3 methylation in Δ*nleE* EPEC-infected cells could be fully restored by wild-type NleE but not the SAM-binding deficient R107A mutant (Figure 7F). Consistently, NleE could completely disrupt the ubiquitin chain-binding ability of Flag-ZRANB3 during EPEC infection, which also required Arg107 in NleE (Figure 7G). Thus, ZRANB3, like TAB2/3, is a bona fide target of NleE methyltransferase activity under physiological conditions.

Overexpression of ZRANB3 in the presence or absence of NleE did not affect NF-κB activation (Figure S8). ZRANB3 has 1, 077 amino acids; its N-terminal half is a helicase domain and the C-terminus harbors multiple domains including the NZF domain. Recent studies suggest that ZRANB3 is localized in nucleus and functions in DNA replication stress response to maintain genome stability [9], [10], [11]. ZRANB3 is recruited to damaged replication forks to promote fork restart. We also observed that EGFP-ZRANB3 was recruited to laser-generated stripes where DNA damage occurred (Figure S9). Notably, co-expression of NleE, which was found distributed in both the cytoplasm and nucleus (Figure S10) and resulted in complete methylation of ZRANB3 (Figure 7B), did not affect ZRANB3 recruitment to DNA damage sites (Figure S9). It has been proposed that damage-induced recruitment of ZRANB3 is mediated by its binding to K63-linked polyubiquitin chains on PCNA, a protein playing a central role in promoting faithful DNA replication [9], [11]. Our results suggest that another structural region in ZRANB3 is more likely responsible for its recruitment to DNA damage sites and the NZF domain-mediated polyubiquitin-chain binding probably participates in other aspects of ZRANB3 function that remains to be defined. It is also worth noting here that the activity of NleE offered us a unique approach to achieve functional disruption of a single domain within a large multiple-domain protein.

## Discussion

NleE is a unique SAM-dependent methyltransferase in catalyzing cysteine methylation. The structure of NleE bears an overall Rossmann-like fold and more resembles that of Class I SAM-dependent methyltransferase, but its SAM-binding mode and conformation are completely different. This indicates an independent evolution of the two sub-lineages within the methyltransferase family and highlights the convergent evolution of bacterial virulence activity. The unique fold of NleE expands the repertoire of SAM-dependent methyltransferases and highlights the convergence on methylation chemistry from different three dimensional folds.

Cysteine methylation is rare; a recent example is methylation of Cys39 in Rps27a, a nonessential yeast ribosomal protein [17]. Rps27a is structurally similar to the N-terminal domain of Ada protein and Cys39 is also one of the four Zn-coordinating cysteines, suggesting a similar non-enzymatic methyl transfer. This supports that Zn coordination facilitates methyl transfer onto the cysteine thiol. The high abundance of zinc finger [18] also indicates that other zinc-finger motifs might be potential methylation targets of some methyltransferases. A recent study on a radical SAM methyltransferase RlmN shows methylation of a cysteine not bound to the zinc [19], [20], further highlighting a chemical diversity of cysteine methylation.

In addition TAB2/3, we now also identify as ZRANB3 as another efficient methylation substrate of NleE (EPEC 2348/69 strain). As NleE homologues are also present in other pathogenic *E. coli* strains as well as *Salmonella* and *Shigella* spp., it is possible that different NleE homologues, produced by different bacterial pathogens, may target different host substrates. NleE efficiently methylates ZRANB3-NZF and abolishes its ubiquitin-chain binding but does not affect ZRANB3 recruitment to DNA damage sites. Proper function of ZRANB3 depends on its interaction with PCNA [9], [10], [11] and three domains, the PCNA-interacting protein motif (PIP-box), the AlkB2 PCNA-interaction motif (APIM), and the NZF domain, are proposed to be involved. These studies are all based on arbitrary deletion of an internal fragment in ZRANB3, which might complicate data interpretation. NleE offers an unprecedented opportunity for specifically inactivating the NZF in ZRANB3 *in situ* without interfering with other domain functions, which reveals a dispensable role of the NZF domain in DNA damage recruitment. Thus, the NZF either plays little role in the recruitment or is functionally redundant to other domains. It is also plausible that NZF-mediated poly-ubiquitin chain binding may regulate the activity of ZRANB3 itself or fulfill functions as yet undefined.

## Materials and Methods

### Plasmids, antibodies and bacteria strains

The cDNA expression constructs for NleE, TAB2/3, TAB2/3-NZF, LUBAC-NZFs and NEMO-NZF were described previously [6]. NleE point mutations were generated by QuickChange Site-Directed Mutagenesis Kit (Stratagene). cDNA for mTrabid was kindly provided by Dr. Paul Evans (University of Sheffield, UK). IMAGE clones for ZRANB2 and ZRANB3 were purchased from Source BioScience LifeSciences Inc. NZFs of Trabid, ZRANB2 and ZRANB3 were PCR-amplified and inserted into the pGEX-6p-2 vector for recombinant expression in *E. coli*, and the full-length ZRANB3 was inserted into pCS2-Flag for transient expression in mammalian cells. pEF-Flag-TAB3ΔNZF-ZRANB2-NZF chimera were constructed as previously described [6], [21]. Luciferase plasmids were also described previously [22]. All the plasmids were verified by DNA sequencing. Antibodies for EGFP (sc8334), GAL4 AD (C-10, sc-1663) and ubiquitin (P4D1, sc8017) were purchased from Santa Cruz; Anti-Flag (M2) antibody, anti-tubulin antibody and EZview Red ANTI-FLAG M2 Affinity Gel were from Sigma. Antibody for c-Myc (9E10, MMS-150R-200) was purchased from Covance. Cell culture products were from Invitrogen, and all other reagents were Sigma-Aldrich products unless noted.

### Protein purification, crystallization and data collection

His-SUMO-NleE was expressed in *E. coli* BL21 (DE3) Gold strain. Se-Met labeled NleE was expressed in *E. coli* B834 (DE3) as previously described [23]. NleE was purified sequentially by nickel affinity chromatography, Ulp1 digestion and Mono Q+ Superdex 75 chromatography. Expression and purification of NleE mutants and GST-NZFs were essentially the same as previously described [6]. Purified NleE was concentrated to 20 mg/ml in a buffer containing 20 mM Tris-HCl (pH 8.0) and 100 mM NaCl. Crystals were grown using vapor-diffusion hanging-drop method at 19°C for one week against a reservoir buffer containing 22% PEG3350 and 0.2 M ammonium citrate dibasic. Se-Met crystals were obtained using Se-Met labeled NleE plus 1 mM SAM against 20% PEG3350, 0.125 M ammonium citrate dibasic and 0.1 M sodium malonate (pH 7.0). Crystals were cryo-protected in the well buffer supplemented with 25% glycerol and flash-freezed in liquid nitrogen. Diffraction data were collected at Shanghai Synchrotron Radiation Facility (SSRF) BL-17U at the wavelength of 0.9789 Å for Se-Met crystals and 0.9792 Å for native crystals. All data were processed in the HKL2000 [24].

### Structure determination and refinement

The phase for NleE was determined from the Se-Met crystal data using the single wavelength anomalous dispersion method [25]. Phasing and initial model building were accomplished using the AutoSol function of PHENIX. Automatic model building was performed using the 2.3-Å native data in PHENIX.Autobuild [26]. The autobuild model was manually adjusted in Coot [27]. The final model was refined in PHENIX.Refine. All the structural figures were prepared using the PyMol program (http://www.pymol.org).

### Cell culture, immunoprecipitation, luciferase assay and fluorescence staining

Mammalian cell culture, transfection, immunoprecipitation and luciferase assays were basically the same as those described previously [6]. Rhodamine-Phalloidin staining of F-actin also follows that described the previous literature [28]. EPEC strains and infection protocols were described previously [6].

### Yeast cell extract preparation

Yeast whole cell extracts were prepared as previously described with some minor modifications [29]. 20 OD_600_ units of yeast cells were harvested and freezed in liquid nitrogen. 600 µl of yeast lysis buffer (1.85 M NaOH and 7.4% β-mercaptoethanol) were added and cells were kept on ice for 10 min. Trichloroacetic acid (TCA) was then added to a final concentration of 25% and cell lysates were incubated on ice for another 10 min. After centrifugation at 4°C for 30 min, the pellet was washed with cold acetone for four times. The air-dried pellet was solubilized in the SDS loading buffer and the supernatant was loaded onto an SDS-PAGE for further immunoblotting analysis.

### Mass spectrometry analyses of NZF methylation by NleE

Flag-TAB2/HOIL-1L-V195R/Sharpin-S346R immunopurified from infected 293T cells, Flag-TAB3/TAB3ΔNZF-ZRANB2-NZF co-expressed with NleE in 293T cells, and GST-Vps36/GST-ZRANB3-NZF treated with NleE are subjected to in-gel trypsin digest and subsequent tandem mass spectrometry analysis similarly as previously described [6]. The V195R and S346R mutations in HOIL-1L and Sharpin, respectively, were introduced to facilitate mass spectrometry identification of the target tryptic peptides.

For mass spectrometry analysis of ZRANB3 methylation by NleE, Flag-ZRANB3 was digested by Glu-C in solution. 293T cells transfected with Flag-ZRANB3-expressing plasmid and infected with EPEC were first harvested in buffer A (50 mM Tris-HCl, pH 7.5, 150 mM NaCl, 20 mM n-octyl-β-D-glucopyranoside (INALCO) and 5% glycerol) supplemented with an EDTA-free protease inhibitor mixture (Roche Molecular Biochemicals). Cells were lysed by ultrasonication. The supernatant was pre-cleared by protein G–Sepharose at 4°C for 1 h and subjected to anti-Flag immunoprecipitation. Following 4-h incubation, the beads were washed once with buffer A and then five times with TBS buffer (50 mM Tris-HCl, pH 7.5, and 150 mM NaCl). Bound proteins were eluted with 600 mg/ml Flag peptide (Sigma) in the TBS buffer. The eluted protein was diluted 8 times with 50 mM Tris-HCl (pH 8.5) and then concentrated to 20∼30 µl using the Vivaspin (30,000 MWCO, 500 µl, Sartorius). Tris-HCl (pH 8.5) and Urea were then added to the final concentrations of 0.1 M and 0.8 M, respectively. Ultrasonication was performed to facilitate solubilization of the denatured proteins. The proteins were reduced in 5 mM TCEP (Tris-(carboxyethyl) phosphine hydrochloride) at 55°C for 20 min and then alkylated in 10 mM iodoacetamide at room temperature in dark for 15 min. The alkylated proteins were digested with the sequencing grade Glu-C (Roche Molecular Biochemicals) at 25°C overnight. An aliquot of peptide solution was analyzed by tandem mass spectrometry as previously described [6].

### Molecular docking of TAB2-NZF into NleE-SAM structure and MD simulation of the docked complex

The NZF domain of TAB2 (PDB code 3A9J) [30] was used to model the NleE-NZF complex. The cysteine residues coordinating the Zn (Cys670, Cys673, Cys684 and Cys687) were deprotonated and hydrogen atoms were added using the Protein Local Optimization Program [31], [32], [33]. Protein-protein docking was carried out using the RosettaDock program (Rosetta 3.1) [34], [35] with distance restraints enforced between the carbon atom of donor methyl group in SAM and the sulfur atoms of Cys673 or Cys687 in NZF (cutoff value of 10 Å). Distance restraints between the Zn and sulfur atoms of the four cysteines were also added to ensure the correct spatial geometry of the zinc finger motif. The docking poses were clustered using the NMRCLUST program [36] according to the RMSD values of Cα atoms of TAB2-NZF using the NleE structure as the reference. Representative models of the largest four clusters were selected for further MD simulation refinement.

All the MD simulations were set up by employing the Gromacs 4.07 package [37] with amber 99SB force filed [38] in the TIP3P explicit water model [39]. The tetrahedron-shaped zinc parameters were applied in the MD simulation [40], [41]. After minimization and equilibration, the production run was performed in NVT for 15 ns (300 K) without positional restraints. The short-range electrostatic and Lennard-Jones interactions in the simulations were calculated using a force-shifted cutoff value of 12 Å and 10 Å, respectively. The Long-range electrostatic interactions were computed by the Particle Mesh Ewald method [42]. The covalent bonds involving hydrogen atoms were constrained with the LINCS algorithm [43]. The non-bound interaction energy between TAB2-NZF and NleE was computed by accounting the sum of electrostatic (E_coul-SR_) and vander Waals (E_LJ-SR_) interaction terms in short range. The surface accessible area is calculated in DSSP program [44], and the related solvent accessibility is measured on the ASA-View Server [45]. The trajectories of last 10-ns MD simulations were saved every 100 ps and further analyzed. The interaction energy between TAB2-NZF and NleE, the RMSD, and the distance of polarized “CH3” group of SAM to the sulfur of Cys673 and Cys687 were measured and compared to obtain the near-native complex structure. The model derived from the cluster 1 of distance restraint sampling was the best model, on which another 15-ns MD simulation was carried out for further optimization.

### *In vitro* methylation and ubiquitin chain pulldown assays

8 µg of GST-TAB2-NZF was incubated with 6 µg of NleE or its mutants (without exogenous SAM) or 2 µg of NleE (with 0.8 mM exogenous SAM) for 30 min at 37°C in 30 µl of buffer containing 50 mM Tris-HCl (pH 7.5), 150 mM NaCl, 5 mM DTT and 0.1% NP-40. The reaction mixtures were separated on a 12% native-PAGE gel, followed by Coomassie blue staining. ^3^H-SAM labeling of different GST-NZFs were carried out as previously described [6]. To examine NleE methylation of TAB2/ZRANB3 *in vivo*, Flag-TAB2/ZRANB3 co-expressed with an empty vector or NleE was immunopurified from 293T cells and subjected to *in vitro* methylation using 0.6 µg of recombinant NleE and 0.55 µCi of ^3^H-SAM. To examine the effect of NleE modification on the ubiquitin-chain binding activity of ZRANB3 *in vitro*, 20 µg of GST-ZRANB3-NZF was incubated with 3 µg of NleE for 30 min at 37°C in a 40-µl reaction containing 0.8 mM SAM. The GST-tagged proteins were then immobilized onto Glutathione Sepharose 4B beads (GE Healthcare) for GST pulldown of Lys48, or Lys63-linked ubiquitin chains or linear tetra-ubiquitin similarly as that described previously [6]. To assay NleE modification and inactivation of cellular ZRANB3, 293T cells, co-transfected with Flag-ZRANB3 and EGFP-NleE as indicated, were harvested and re-suspended in 50 mM Tris-HCl (pH 7.5), 150 mM NaCl, 0.1% Triton X-100 and 5% glycerol. Cells were lysed by ultrasonication. The supernatant was pre-cleared using Protein G Sepharose (GE Healthcare) and then subjected to overnight pulldown by Lys63-linked ubiquitin chains or SBP (streptavidin binding peptide)-tagged linear tetra-ubiquitin chains [6].

### Assay of ZRANB3 recruitment to DNA damage sites

U2OS cells cultured in 35-mm glass bottom culture dish (MatTek) were co-transfected with EGFP-ZRANB3 and RFP-NleE expression plasmids as indicated by using the Vigofect reagent (Vigorous). Cells were sensitized by addition of 10 µM BrdU for 16 h and then transferred to the environmental chamber (5% CO_2_, 37°C) in the spinning disk confocal imaging system (PerkinElmer UltraVIEW VOX). Following visualization under Nikon Eclipse Ti inverted microscope, cells with both EGFP and RFP fluorescence were subjected to laser microirradiation using the FRAP (Fluorescence recovery after photobleaching) module and live images were then taken at indicated times points after the microirradiation.

### Accession numbers

The coordinates of the NleE structure together with the structure factors have been deposited in the Protein Data Bank with the accession code 4R29.

## Supporting Information

Figure S1 **The K181A mutation facilitates crystal contacts in NleE structure.** *Left*: A complete unit cell (C2, 4 molecules per asymmetric unit) of the NleE crystal is shown as green monoclinic prism; protein chains in the crystal are shown as lines with color scheme indicated on the lower right. The mutated epitopes involved in close crystal contacts are circled with black dotted line. *Right*: an enlarged ribbon-diagram view of one crystal contact involving the K181A epitope.
(TIF)

Figure S2 **Analysis of the NleE assembly in the asymmetric unit (ASU) of the crystal.** (A) The overall structure of NleE tetramer in the ASU. The four NleE chains (A–D) are colored in green, cyan, purple and yellow, respectively (the chain ID applies to all panels in this figure). (B) The solvent accessible surface area of different chains and the buried areas resulting from the tetramer formation. (C) A list of all predicted stable assemblies in solution that can make the crystal. ΔG^int^ and ΔG^diss^ indicate the solvation free energy gained upon formation of the assembly and the free energy of assembly dissociation, respectively. The stability of the NleE tetramer complex presented in the ASU was also analyzed and the results are appended at the bottom of the table. All the calculations and predictions in (B) and (C) were performed using the PISA program (http://www.ebi.ac.uk/pdbe/pisa/).
(TIF)

Figure S3 **Computational analyses of Cys673 in TAB2-NZF being the specific methylation site by NleE.** (A) The overall structure of TAB2-NZF domain (PDB ID code: 3A9J). The NH-S hydrogen bonds are shown as black dashed lines and the Zn is shown as red dot. (B) The solvent accessible surface area of Cys670, Cys673, Cys684 and Cys687 in TAB2-NZF structure. (C) The dynamic motion (RMSD), the interaction energy and the distance between Sγ of Cys673/Cys687 and Cε of SAM of the largest four clusters in Cys673/Cys687 restrained the 15-ns MD simulation analysis.
(TIF)

Figure S4 **Yeast two-hybrid assay of NleE interaction with TAB3 truncations.** (A, C) Yeast strain AH109 was transformed with plasmid combinations as illustrated (bait+prey). Plasmid combinations resulting in a positive interaction are colored in red. (B) Expression of TAB3 truncations in yeast. Shown are immunoblots of GAL4 AD-fused TAB3 truncation proteins, GAL4 BD-Myc-NleE and tubulin. Number denotation is the same as that in (A). *, a nonspecific band from the long exposure.
(TIF)

Figure S5 **Mass spectrometry analysis of NleE modification of a chimeric TAB3.** TAB3ΔNZF-ZRANB2-NZF is a chimeric construct with replacement of the NZF domain in TAB3 with that from ZRANB2. Flag-tagged TAB3 or the chimeric TAB3 was co-expressed with or without NleE in 293T cells and subjected to Flag-immunoprecipitation and further mass spectrometry analysis. Shown are the extracted ion chromatograms of triply charged TAB3-NZF (upper panel) and doubly charged ZRANB2-NZF peptide containing the corresponding cysteine (lower panel). The unmethylated peptides are shown in blue trace and the methylated ones are in red with the methylated cysteine residue in red. C_ca_, carbamidomethylated cysteine generated from iodoacetamide treatment during sample preparation; C_me_, NleE-methylated cysteine.
(TIF)

Figure S6 **Multiple sequence alignment of the NZF motifs.** Alignment was generated in the GeneDoc program. The name of NZF motif was indicated on the left to the sequence. The amino acid sequence of the motif is derived from human protein unless indicated in the parentheses. Conserved residues are in grey. The starting and ending residue numbers for each NZF is shown on the left and right of the sequence, respectively. The four zinc-coordinating cysteines are strictly conserved and highlighted in yellow. NleE-methylated cysteine in TAB2/TAB3-NZF, Vsp36-NZF and ZRANB3-NZF is shown in red with green background.
(TIF)

Figure S7 **Tandem mass (MS/MS) spectra of the triply charged peptides derived from NleE-treated Vps36-NZF (upper panel) and ZRANB3-NZF domain (lower panel).** The b- and y-type product ions are marked in the spectrum and also illustrated along the peptide sequence shown on top of the spectrum, which unambiguously identifies the second cysteine as the methylated residue. The rest of three non-methylated cysteines were carbamidomethylated due to the iodoacetamide treatment during sample preparation as described in the method section.
(TIF)

Figure S8 **Luciferase assays of ZRANB3 and its modification by NleE on NF-κB activation.** 293T cells were transfected with indicated amount of TAB3 or ZRANB3 expression plasmids together an empty vector or NleE plasmid. Y axis is on the logarithmic scale. Error bars indicate standard deviation. Experiments were performed at least three times with similar results obtained.
(TIF)

Figure S9 **Effects of NleE expression on ZRANB3 recruitment to DNA damage sites.** U2OS cells transfected with EGFP-ZRANB3 together with RFP or RFP-NleE were sensitized by 10 µM BrdU for 16 h prior to laser microirradiation. Shown are fluorescence images taken at indicated time points after the microirradiation. Experiments were performed for at least three times with similar results obtained.
(TIF)

Figure S10 **Localization of NleE in HeLa cells.** HeLa cells were transfected with EGFP-NleE plasmid. Shown are fluorescence images of the transfected cells. DAPI and Rhodamine-Phalloidin stain the nuclei (blue) and F-actin (red), respectively.
(TIF)

Table S1 **Crystal data collection and refinement statistics.**
(DOCX)

Table S2 **Summary of zinc finger (ZF) substrates profiling for NleE.** A survey of Zinc Finger-containing proteins for methylation by recombinant NleE *in vitro*. The DNA encoding the indicated ZF regions of each protein was isolated by PCR and cloned into appropriate plasmid vectors, and the proteins were expressed individually and purified as GST- or His-fusion proteins in *E. coli*. Each purified protein was incubated under standard reaction conditions with recombinant NleE enzyme and ^3^H-SAM as described in Materials and Methods followed by gel electrophoresis and autoradiography. The relative ability of NleE to methylate each protein is indicated by the plus (high reactivity) or minus (no reactivity) signs relative to TAB2. Each finger protein encompassed the complete ZF region and included at least 2 amino acids N- and C-terminal to the Cys (or His) Zn^2+^ coordination residues. For the non-C4 ZF proteins, the class of ZF is indicated in parentheses. The amino acid sequence utilized in each construct and complete cloning details for each fusion protein are available upon request.
(DOCX)
