# Supplementary figures and images for: Structure and Specificity of the Bacterial Cysteine Methyltransferase Effector NleE Suggests a Novel Substrate in Human DNA Repair Pathway
Source: PLoS Pathog. 2014 Nov 20;10(11):e1004522. doi: 10.1371/journal.ppat.1004522 (PMC4239114; doi:10.1371/journal.ppat.1004522)

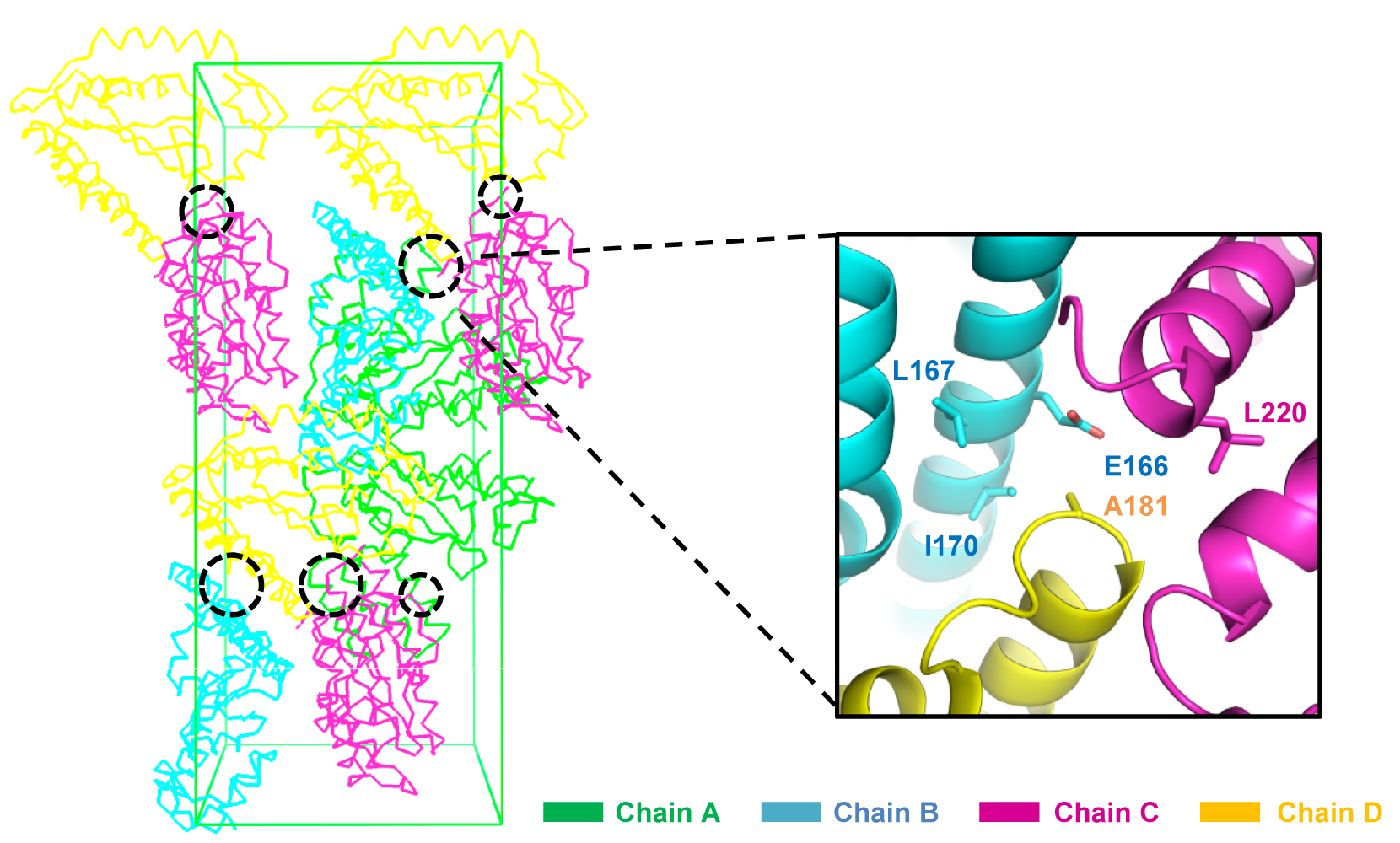

Supplement: Figure S1 — The K181A mutation facilitates crystal contacts in NleE structure. Left: A complete unit cell (C2, 4 molecules per asymmetric unit) of the NleE crystal is shown as green monoclinic prism; protein chains in the crystal are shown as lines with color scheme indicated on the lower right. The mutated epitopes involved in close crystal contacts are circled with black dotted line. Right: an enlarged ribbon-diagram view of one crystal contact involving the K181A epitope. (TIF) [file ppat.1004522.s001.tif]

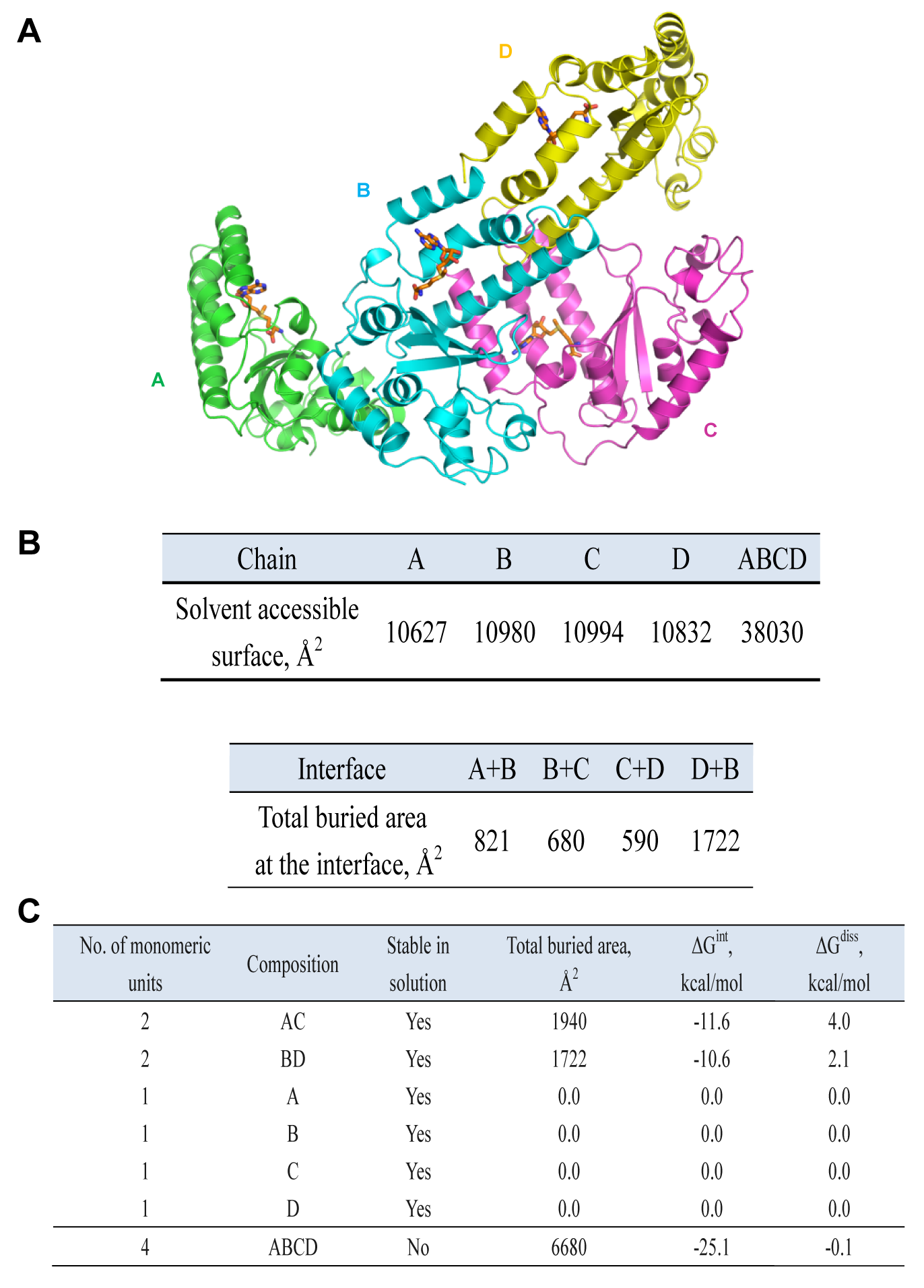

Supplement: Figure S2 — Analysis of the NleE assembly in the asymmetric unit (ASU) of the crystal. (A) The overall structure of NleE tetramer in the ASU. The four NleE chains (A–D) are colored in green, cyan, purple and yellow, respectively (the chain ID applies to all panels in this figure). (B) The solvent accessible surface area of different chains and the buried areas resulting from the tetramer formation. (C) A list of all predicted stable assemblies in solution that can make the crystal. ΔGint and ΔGdiss indicate the solvation free energy gained upon formation of the assembly and the free energy of assembly dissociation, respectively. The stability of the NleE tetramer complex presented in the ASU was also analyzed and the results are appended at the bottom of the table. All the calculations and predictions in (B) and (C) were performed using the PISA program (http://www.ebi.ac.uk/pdbe/pisa/). (TIF) [file ppat.1004522.s002.tif]

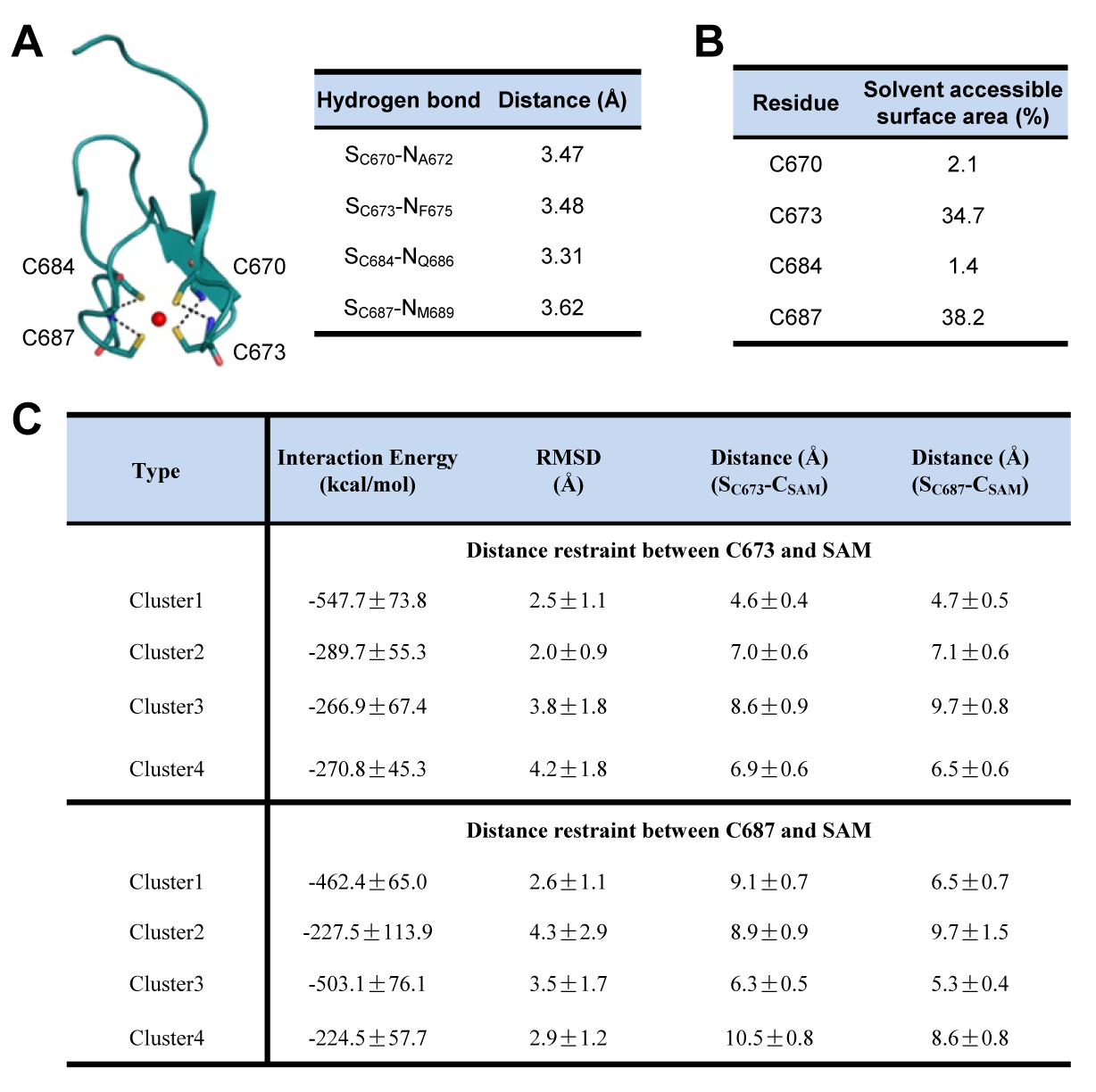

Supplement: Figure S3 — Computational analyses of Cys673 in TAB2-NZF being the specific methylation site by NleE. (A) The overall structure of TAB2-NZF domain (PDB ID code: 3A9J). The NH-S hydrogen bonds are shown as black dashed lines and the Zn is shown as red dot. (B) The solvent accessible surface area of Cys670, Cys673, Cys684 and Cys687 in TAB2-NZF structure. (C) The dynamic motion (RMSD), the interaction energy and the distance between Sγ of Cys673/Cys687 and Cε of SAM of the largest four clusters in Cys673/Cys687 restrained the 15-ns MD simulation analysis. (TIF) [file ppat.1004522.s003.tif]

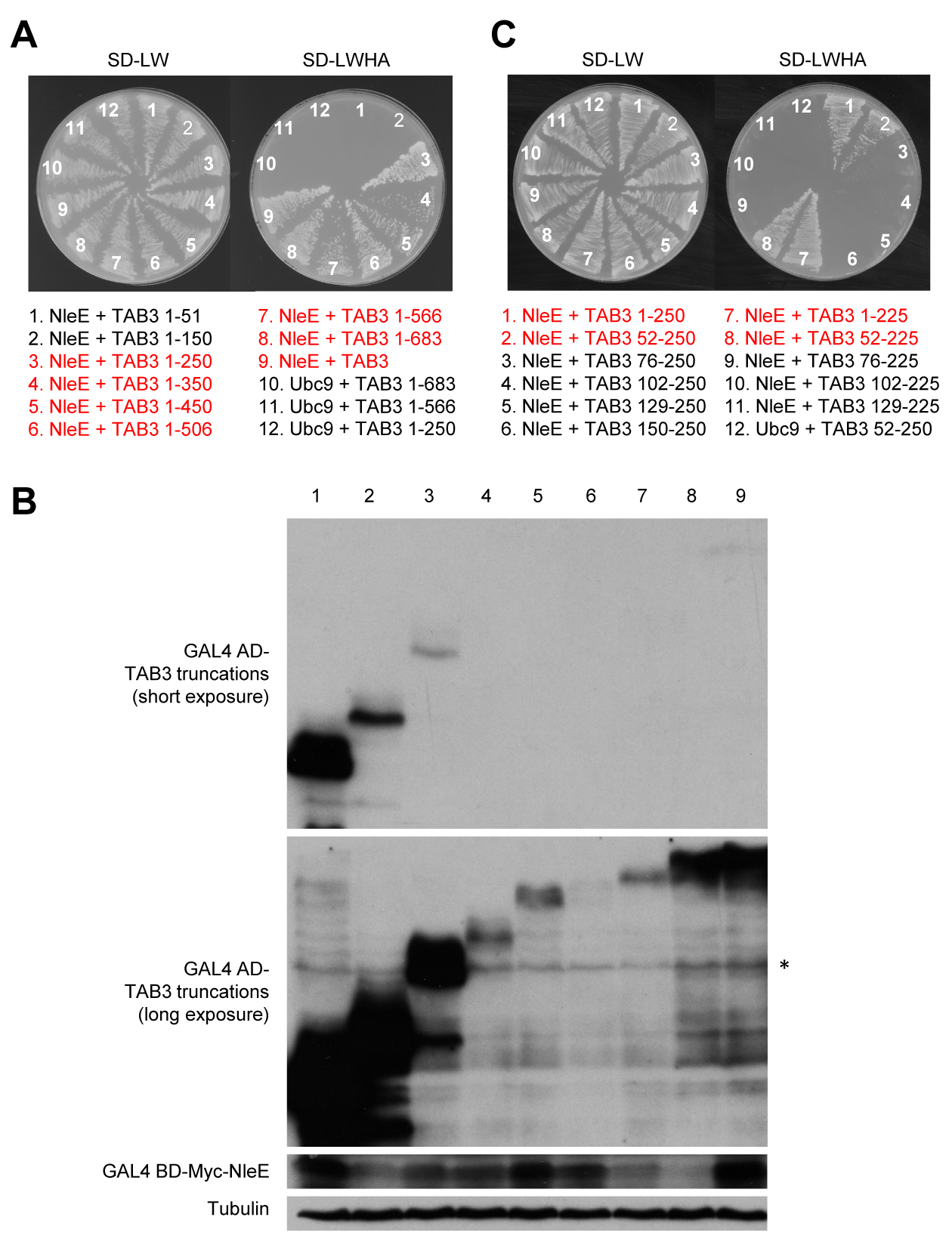

Supplement: Figure S4 — Yeast two-hybrid assay of NleE interaction with TAB3 truncations. (A, C) Yeast strain AH109 was transformed with plasmid combinations as illustrated (bait+prey). Plasmid combinations resulting in a positive interaction are colored in red. (B) Expression of TAB3 truncations in yeast. Shown are immunoblots of GAL4 AD-fused TAB3 truncation proteins, GAL4 BD-Myc-NleE and tubulin. Number denotation is the same as that in (A). *, a nonspecific band from the long exposure. (TIF) [file ppat.1004522.s004.tif]

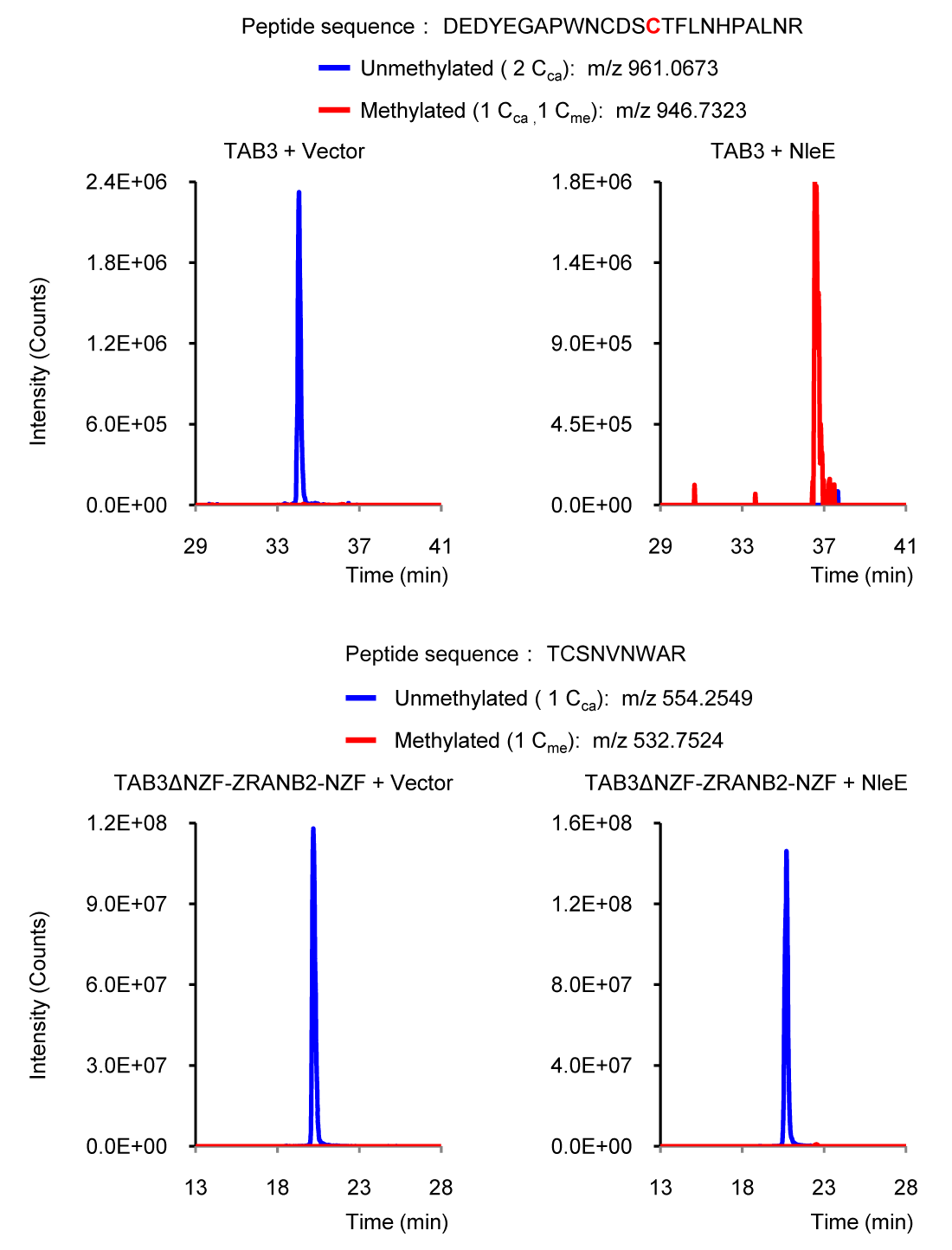

Supplement: Figure S5 — Mass spectrometry analysis of NleE modification of a chimeric TAB3. TAB3ΔNZF-ZRANB2-NZF is a chimeric construct with replacement of the NZF domain in TAB3 with that from ZRANB2. Flag-tagged TAB3 or the chimeric TAB3 was co-expressed with or without NleE in 293T cells and subjected to Flag-immunoprecipitation and further mass spectrometry analysis. Shown are the extracted ion chromatograms of triply charged TAB3-NZF (upper panel) and doubly charged ZRANB2-NZF peptide containing the corresponding cysteine (lower panel). The unmethylated peptides are shown in blue trace and the methylated ones are in red with the methylated cysteine residue in red. Cca, carbamidomethylated cysteine generated from iodoacetamide treatment during sample preparation; Cme, NleE-methylated cysteine. (TIF) [file ppat.1004522.s005.tif]

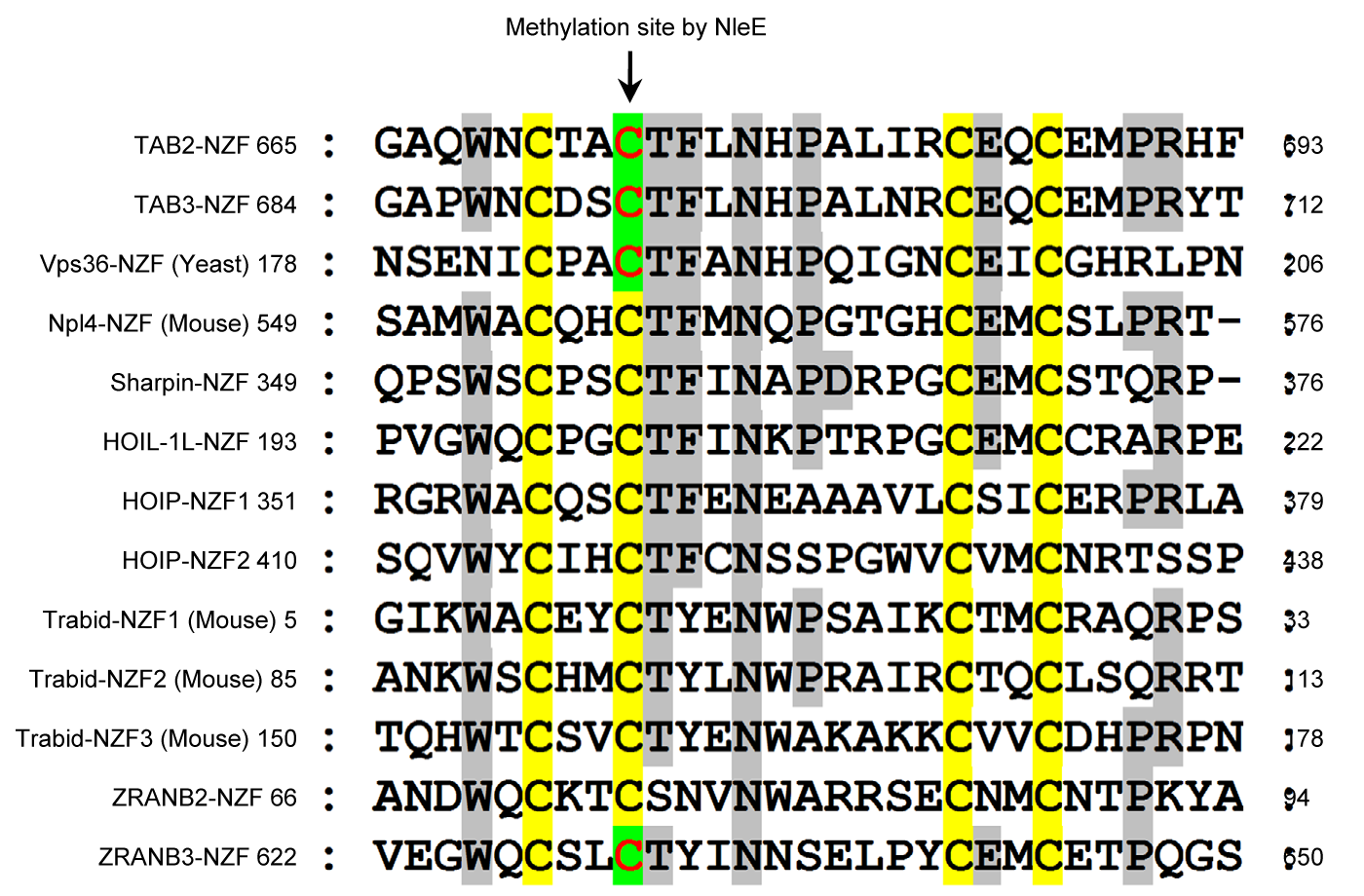

Supplement: Figure S6 — Multiple sequence alignment of the NZF motifs. Alignment was generated in the GeneDoc program. The name of NZF motif was indicated on the left to the sequence. The amino acid sequence of the motif is derived from human protein unless indicated in the parentheses. Conserved residues are in grey. The starting and ending residue numbers for each NZF is shown on the left and right of the sequence, respectively. The four zinc-coordinating cysteines are strictly conserved and highlighted in yellow. NleE-methylated cysteine in TAB2/TAB3-NZF, Vsp36-NZF and ZRANB3-NZF is shown in red with green background. (TIF) [file ppat.1004522.s006.tif]

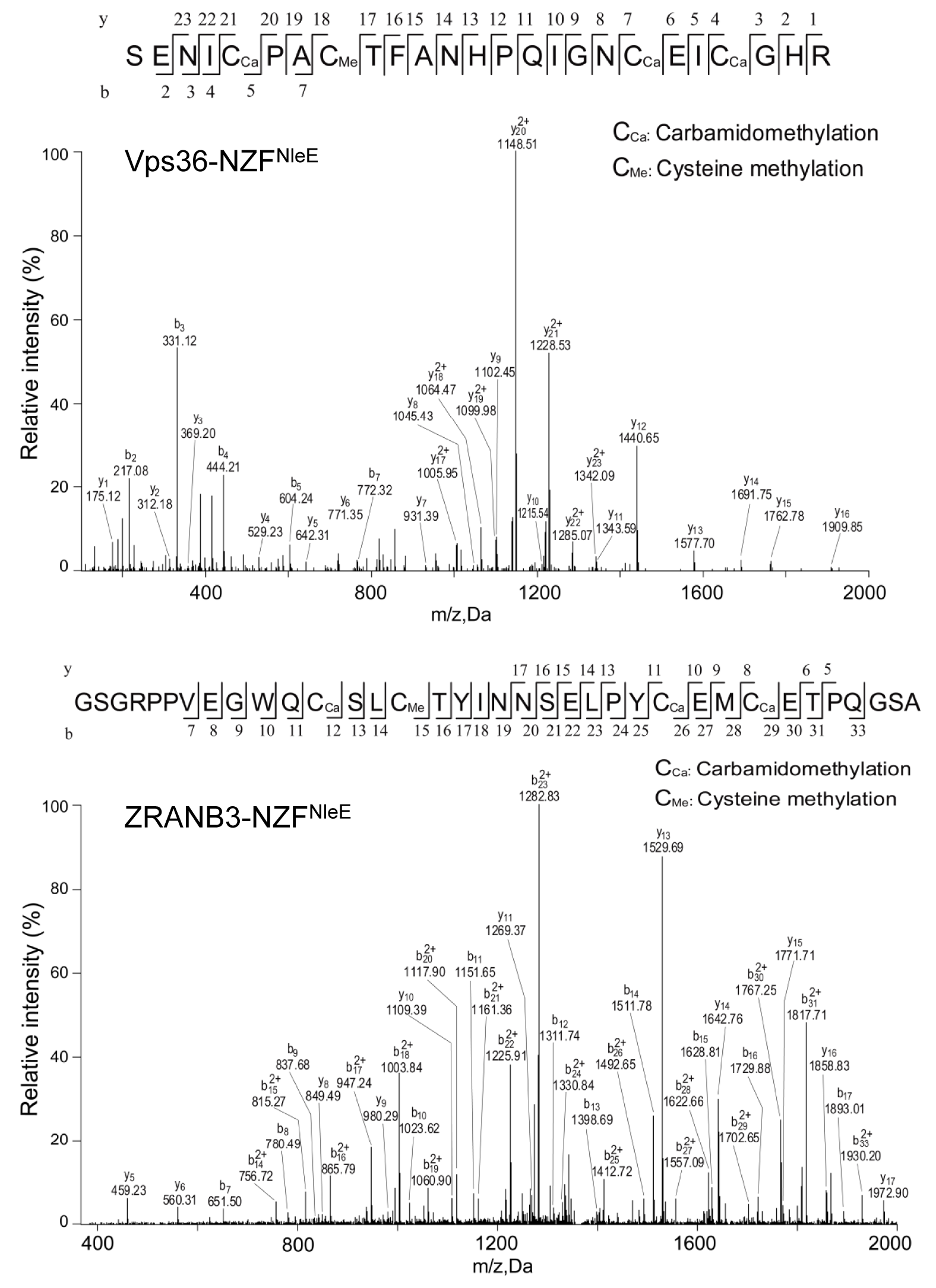

Supplement: Figure S7 — Tandem mass (MS/MS) spectra of the triply charged peptides derived from NleE-treated Vps36-NZF (upper panel) and ZRANB3-NZF domain (lower panel). The b- and y-type product ions are marked in the spectrum and also illustrated along the peptide sequence shown on top of the spectrum, which unambiguously identifies the second cysteine as the methylated residue. The rest of three non-methylated cysteines were carbamidomethylated due to the iodoacetamide treatment during sample preparation as described in the method section. (TIF) [file ppat.1004522.s007.tif]

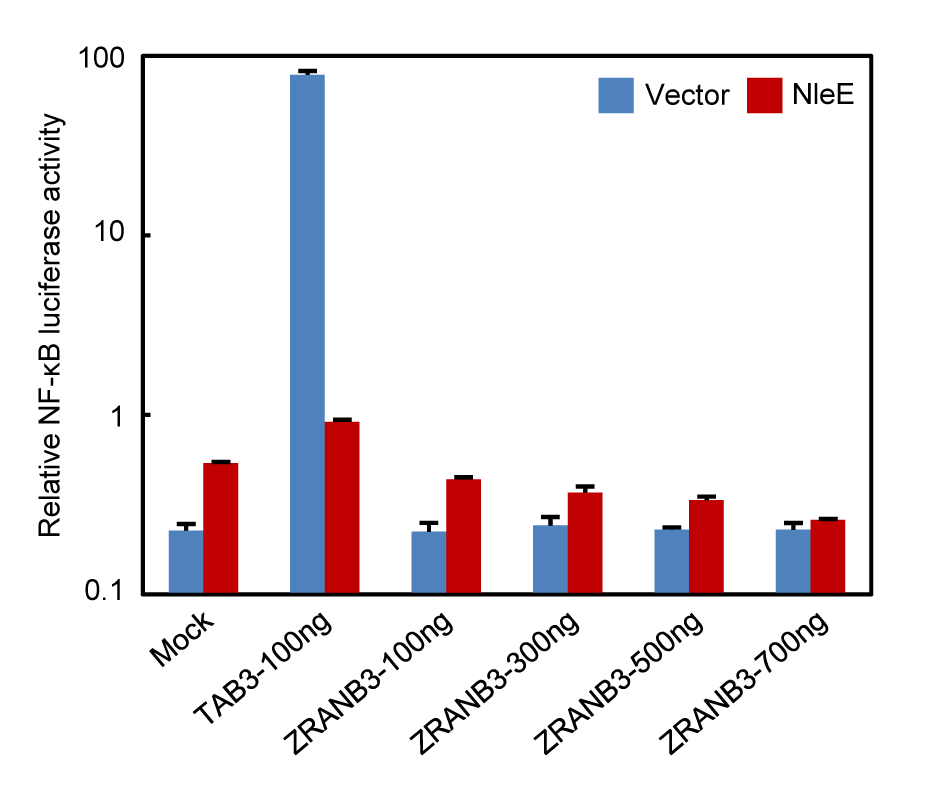

Supplement: Figure S8 — Luciferase assays of ZRANB3 and its modification by NleE on NF-κB activation. 293T cells were transfected with indicated amount of TAB3 or ZRANB3 expression plasmids together an empty vector or NleE plasmid. Y axis is on the logarithmic scale. Error bars indicate standard deviation. Experiments were performed at least three times with similar results obtained. (TIF) [file ppat.1004522.s008.tif]

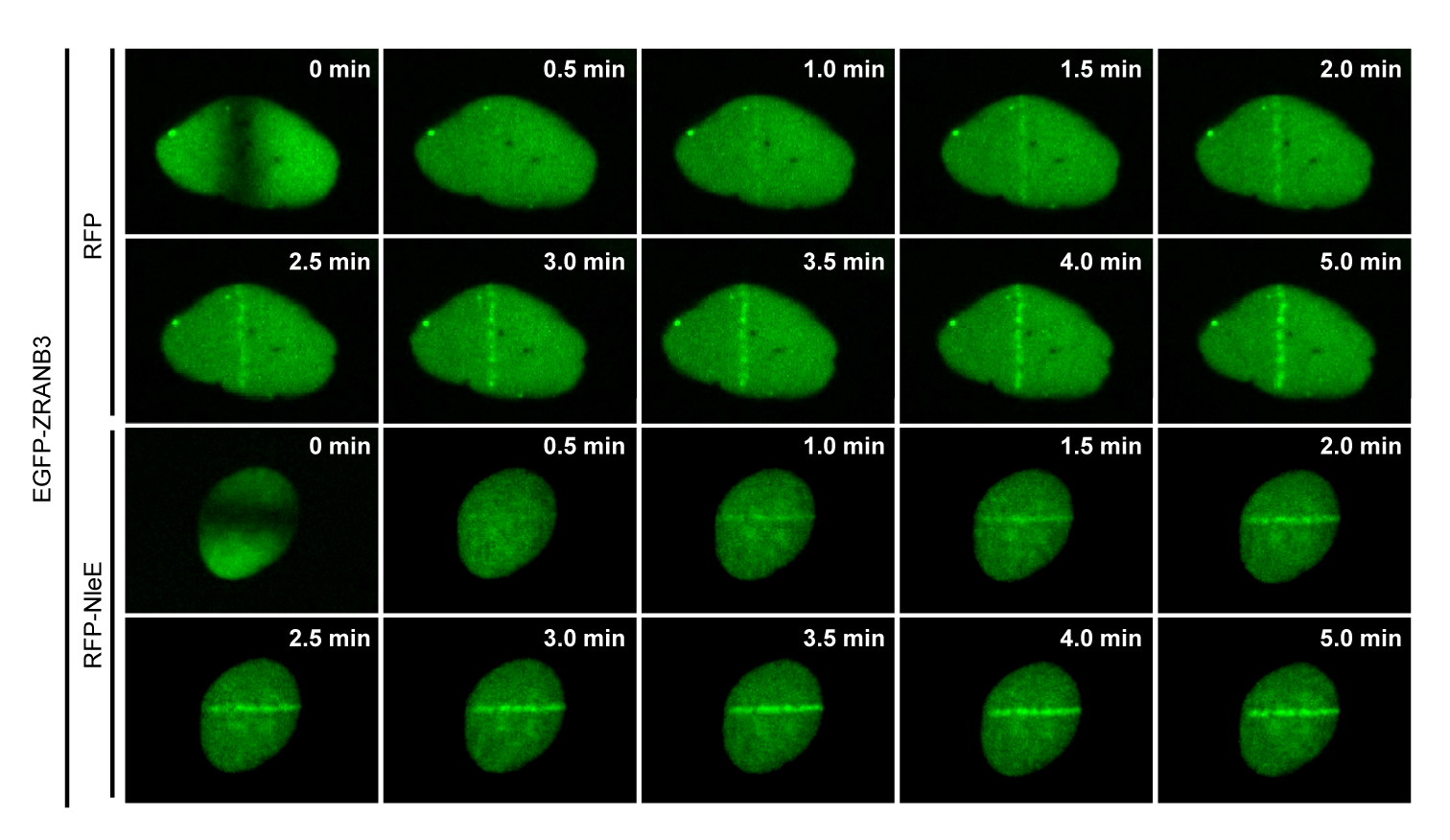

Supplement: Figure S9 — Effects of NleE expression on ZRANB3 recruitment to DNA damage sites. U2OS cells transfected with EGFP-ZRANB3 together with RFP or RFP-NleE were sensitized by 10 µM BrdU for 16 h prior to laser microirradiation. Shown are fluorescence images taken at indicated time points after the microirradiation. Experiments were performed for at least three times with similar results obtained. (TIF) [file ppat.1004522.s009.tif]

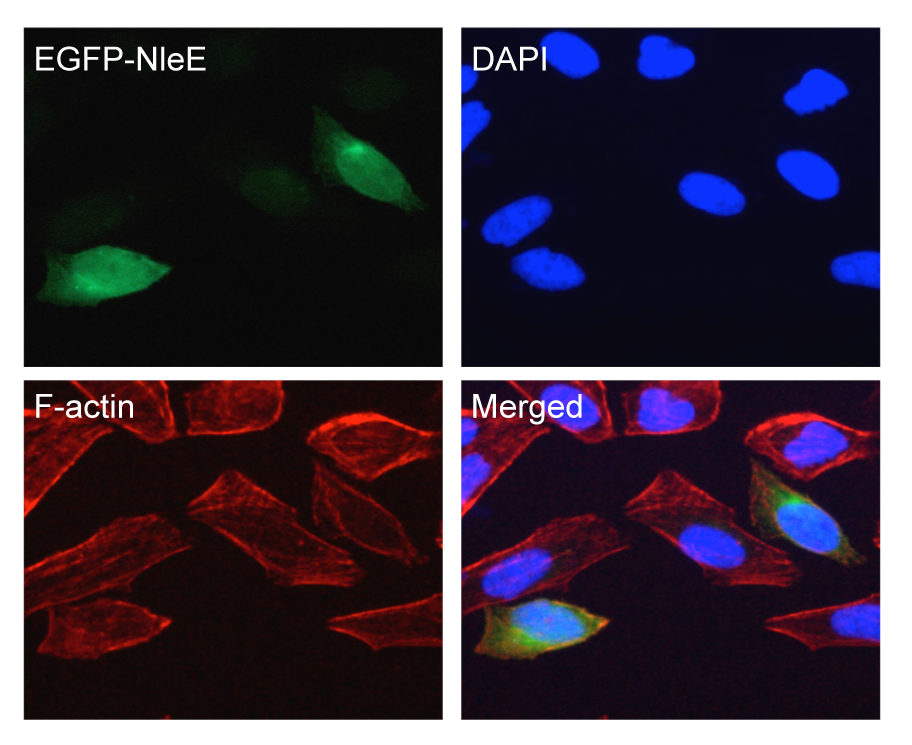

Supplement: Figure S10 — Localization of NleE in HeLa cells. HeLa cells were transfected with EGFP-NleE plasmid. Shown are fluorescence images of the transfected cells. DAPI and Rhodamine-Phalloidin stain the nuclei (blue) and F-actin (red), respectively. (TIF) [file ppat.1004522.s010.tif]
