# Supplementary material for: Structure and Specificity of the Bacterial Cysteine Methyltransferase Effector NleE Suggests a Novel Substrate in Human DNA Repair Pathway
Source: PLoS Pathog. 2014 Nov 20;10(11):e1004522. doi: 10.1371/journal.ppat.1004522 (PMC4239114; doi:10.1371/journal.ppat.1004522)
Supplement: Table S1 — Crystal data collection and refinement statistics. (DOCX) [file ppat.1004522.s011.docx]

| **Crystals** | **Se-Met-NleE** | **Native NleE** |
| --- | --- | --- |
| **Data collection** |  |  |
| Space group | C2 | C2 |
| Wavelength (Å) | 0.9789 | 0.9792 |
| a, b, c (Å) | 134.39, 53.86, 134.66 | 134.30, 53.98, 134.79 |
| α, β, γ (**º)** | 90, 90.16, 90 | 90, 90.11, 90 |
| Resolution range (Å)* | 25-2.60 (2.64-2.60) | 20-2.30 (2.34-2.30) |
| No. of unique reflections | 29,792 (1,450) | 42,433(2,105) |
| Completeness (%) | 99.9 (99.9) | 99.8 (99.0) |
| Redundancy | 7.3 (6.1) | 3.7 (3.4) |
| I/σI | 20.18 (2.0) | 16.9 (3.1) |
| *R*_mege_ (%) | 12.6 (84.5) | 6.9 (27.0) |
| **Refinement statistics** |  |  |
| R_work_/*R*_free_ (%)^†^ |  | 19.7/23.4 |
| No. of protein atoms |  | 6,700 |
| No. of waters |  | 295 |
| Bond lengths (Å) |  | 0.008 |
| Bond angles (**º)** |  | 1.108 |
| **Ramachandran plot statistics** |  |  |
| Most favored regions (%) |  | 95.45 |
| Additional allowed regions (%) |  | 4.29 |
| Outlier region (%) |  | 0.25 |

* The data for the highest resolution shell are shown in parentheses.

^†^ *R*_free_ is calculated by omitting 5% of the total number of reflections in model refinement.
