# Supplementary material for: Structure and Specificity of the Bacterial Cysteine Methyltransferase Effector NleE Suggests a Novel Substrate in Human DNA Repair Pathway
Source: PLoS Pathog. 2014 Nov 20;10(11):e1004522. doi: 10.1371/journal.ppat.1004522 (PMC4239114; doi:10.1371/journal.ppat.1004522)
Supplement: Table S2 — Summary of zinc finger (ZF) substrates profiling for NleE. A survey of Zinc Finger-containing proteins for methylation by recombinant NleE in vitro. The DNA encoding the indicated ZF regions of each protein was isolated by PCR and cloned into appropriate plasmid vectors, and the proteins were expressed individually and purified as GST- or His-fusion proteins in E. coli. Each purified protein was incubated under standard reaction conditions with recombinant NleE enzyme and 3H-SAM as described in Materials and Methods followed by gel electrophoresis and autoradiography. The relative ability of NleE to methylate each protein is indicated by the plus (high reactivity) or minus (no reactivity) signs relative to TAB2. Each finger protein encompassed the complete ZF region and included at least 2 amino acids N- and C-terminal to the Cys (or His) Zn2+ coordination residues. For the non-C4 ZF proteins, the class of ZF is indicated in parentheses. The amino acid sequence utilized in each construct and complete cloning details for each fusion protein are available upon request. (DOCX) [file ppat.1004522.s012.docx]

| **Zinc fingers** | **Methylation by NleE** | **Zinc fingers** | **Methylation by NleE** |
| --- | --- | --- | --- |
| TAB2 | ++++ | TEX13A | - |
| TAB3 | ++++ | NEIL3 ZF1-3 | - |
| Vps36 | ++++ | NUP153 ZF1-4 | - |
| ZRANB3 | ++++ | A20 ZF1-6 | - |
| HOIL-1L | - | CEZANNE | - |
| NPL4 | +/- | CALPAIN15 ZF1-4 | - |
| Sharpin | - | RPS27a | - |
| ZRANB2 ZF1-2 | - | TRX-2 | - |
| ZRANB1 ZF1 | +/- | SPARTAN | - |
| ZRANB1 ZF2 | - | DNA POLκ ZF1-2 | - |
| ZRANB1 ZF3 | + | p53 | - |
| RANBP2 ZF1-8 | - | HIV-1 NCp7 | - |
| MDM2 | - | HIV-2 NCp7 | - |
| MDM4 | - | FOG1 ZF1, 9 (CCHC) | - |
| MDMX | - | KAP1-RBCC (RING, B-BOX) | - |
| YAF2 | - | KAP1-PHD (PHD) | - |
| EWS1 | - | LIMD1 (LIM) | - |
| TLS | - | LIMD2 (LIM) | - |
| RBM10 | - | SNAIL (C2H2) | - |
| RBM5 | - | ZBRK (C2H2) | - |
| RBP56 | - | SZF1 (C2H2) | - |
